# Supplementary figures and images for: Phosphorus flow analysis of different crops in Dongying District, Shandong Province, China, 1995–2016
Source: PeerJ. 2022 Apr 19;10:e13274. doi: 10.7717/peerj.13274 (PMC9029382; doi:10.7717/peerj.13274)

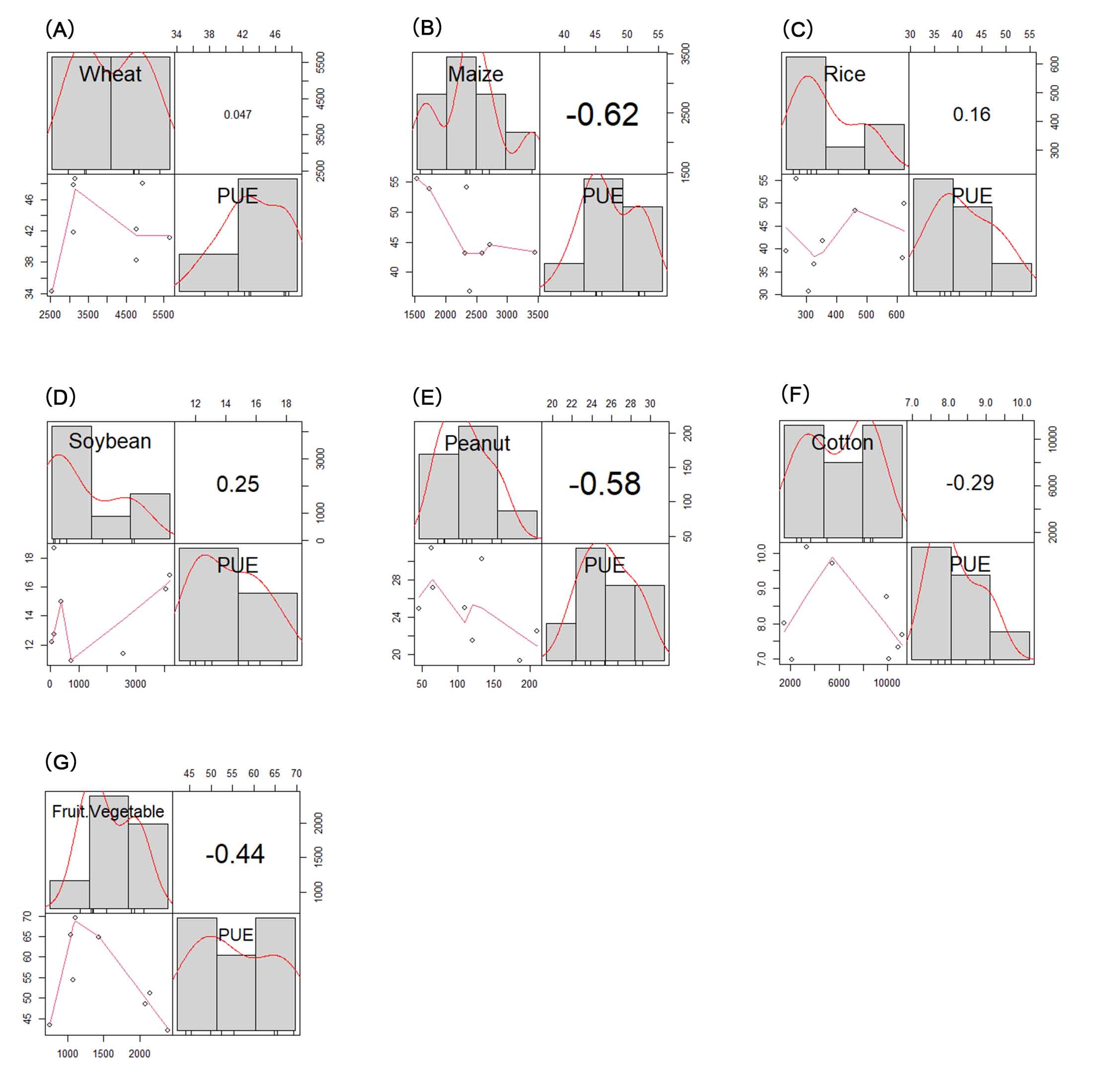

Supplement: Supplemental Information 3 — Pearson correlation coefficients (r) are in the right upper box. [file peerj-10-13274-s003.jpg]
